# Non-Dilated Left Ventricular Cardiomyopathy: Exploring the Spectrum from Phenotype to Disease Entity

**DOI:** 10.3390/jcm15187214

**Published:** 2026-09-17

**Authors:** Francesco Mangini, Massimo Grimaldi, Antonio Di Monaco, Fabrizio Fortunato, Nicola Vitulano, Federico Quadrini, Ilaria Dentamaro, Marco Guglielmo, Francesco Spinelli, Valentina Grimaldi, Francesca Musella, Luca Sgarra, Simona Quarta, Sergio Suma, Robert W. W. Biederman, Roberto Calbi

**Affiliations:** 1 Cardiology Department, Ecclesiastical Entity Regional General Hospital “F. Miulli”, 70021 Acquaviva delle Fonti, BA, Italy; 2 Cardiology Department, Policlinico di Bari University Hospital, 70124 Bari, Italy; 3 Cardiology Department, University Medical Center Utrecht, 3584 CX Utrecht, The Netherlands; 4 Radiology Department, Ecclesiastical Entity Regional General Hospital “F. Miulli”, 70021 Acquaviva delle Fonti, BA, Italy; 5 Cardiology Department, Santa Maria delle Grazie Hospital, 80078 Naples, Italy; 6 Department of Cardiology, Parma University Hospital, 43126 Parma, Italy; 7 Department of Cardiology, West Virginia University, Morgantown, WV 26506, USA; 8 Department of Cardiology, Medical University of South Carolina, Charleston, SC 29425, USA; 9 Department of Cardiology, Roper/Saint Francis Hospital, Charleston, SC 29403, USA

**Keywords:** non-dilated left ventricular cardiomyopathy, cardiomyopathies, arrhythmogenic cardiomyopathy, cardiac magnetic resonance, sudden cardiac death

## Abstract

Non-dilated left ventricular cardiomyopathy has recently emerged as a distinct morpho-functional phenotype defined by non-ischemic left ventricular scarring or fatty replacement or by isolated global left ventricular hypokinesia without scarring in the absence of ventricular dilatation. Whether it represents a distinct biological entity or a shared phenotypic expression across cardiomyopathies remains unresolved. This narrative review examines evidence from genetics, multimodality imaging, natural history, and outcomes. Overlap with dilated and arrhythmogenic cardiomyopathies is extensive: no currently validated genetic, imaging, histological, or circulating marker uniquely identifies the phenotype, and longitudinal studies describe heterogeneous trajectories from reverse remodeling to dilated or predominantly arrhythmogenic evolution. Genetics argues in both directions, as the genes involved are shared yet their distribution differs, pro-arrhythmogenic variants being more frequent than in dilated cardiomyopathy. We propose a hypothesis-generating framework of three probable trajectories, evolving dilated cardiomyopathy, evolving arrhythmogenic cardiomyopathy, or stable non-dilated disease, integrating genotype, multiparametric cardiac magnetic resonance, longitudinal remodeling, and arrhythmic phenotype.

## 1. Introduction

The 2023 European Society of Cardiology (ESC) Guidelines, adopting a morpho-functional phenotype-based classification, for the first time introduced the non-dilated left ventricular cardiomyopathy (NDLVC) phenotype. NDLVC is defined by the presence of non-ischemic left ventricular (LV) scarring or fatty replacement, regardless of global or regional wall motion abnormalities, or by isolated global LV hypokinesia without scarring in the absence of LV dilatation and not explained solely by coronary artery disease or abnormal loading conditions such as hypertension or valvular disease; the phenotype may be further characterized by the presence or absence of systolic dysfunction [1]. However, this definition should be interpreted within the broader context of contemporary cardiomyopathy classifications, which are not uniform, differing in both their conceptual approach and the timing of their updates [1,2,3,4,5]. Consequently, a patient with identical clinical and imaging features may be classified differently depending on the classification framework applied.

To illustrate, a single clinical case is interpreted through four widely used frameworks, taken here as representative examples: the ESC 2023 Guidelines, the Padua Criteria, the Heart Rhythm Society (HRS) Expert Consensus, and the American Heart Association (AHA) Scientific Statement. A 39-year-old man with a family history of premature sudden cardiac death (SCD) in a first-degree relative was evaluated after documentation of non-sustained ventricular tachycardia (NSVT) with right bundle branch block (RBBB) morphology on ambulatory electrocardiographic (ECG) monitoring. Invasive coronary angiography excluded obstructive coronary artery disease. Echocardiography showed a non-dilated left ventricle of normal wall thickness with normal diastolic function. Cardiac magnetic resonance (CMR) showed mildly reduced left ventricular ejection fraction (LVEF, 43%), regional LV wall motion abnormalities, and subepicardial late gadolinium enhancement (LGE) of the basal inferior and inferolateral segments; the right ventricle (RV) was normal in volume and function. Genetic testing identified a pathogenic *DES* variant. This patient is interpreted through the following classification frameworks:

- 2023 ESC Guidelines: NDLVC. The patient fulfills the NDLVC phenotype based on non-ischemic LV scar without LV dilatation, with regional wall motion abnormalities and mildly reduced LVEF further characterizing the phenotype as NDLVC with systolic dysfunction.
- Padua Criteria (2020, refined by the 2023 European Task Force report published in 2024): Arrhythmogenic left ventricular cardiomyopathy (ALVC). The patient fulfills morpho-functional (regional LV wall motion abnormality with mild LV systolic dysfunction), tissue characterization (subepicardial non-ischemic LGE), and arrhythmic (NSVT with RBBB morphology) criteria, consistent with left-dominant ALVC. In the absence of RV involvement, the 2020 Padua Criteria require demonstration of a pathogenic arrhythmogenic cardiomyopathy (ACM) gene variant for a diagnosis of ALVC; the pathogenic *DES* variant therefore constitutes a mandatory major criterion rather than merely supportive etiological evidence [2,3].
- 2006 AHA Scientific Statement: No fitting phenotypic category. The AHA scheme reserves its primary genetic subgroup to an enumerated list that excludes a non-dilated, non-hypertrophic phenotype, assigning dilated and restrictive phenotypes, and desmin among the genes underlying dilated cardiomyopathy to the mixed genetic and non-genetic group. A pathogenic *DES* variant with secondary causes excluded therefore establishes a primary cardiomyopathy of genetic etiology for which this classification offers no fitting phenotypic label.
- 2019 HRS Expert Consensus: Left-dominant ACM. The combination of non-ischemic LV scar, NSVT, preserved RV structure and function, and a pathogenic *DES* variant is consistent with a left-dominant ACM phenotype within the HRS arrhythmogenic cardiomyopathy spectrum.

**Figure 1:**
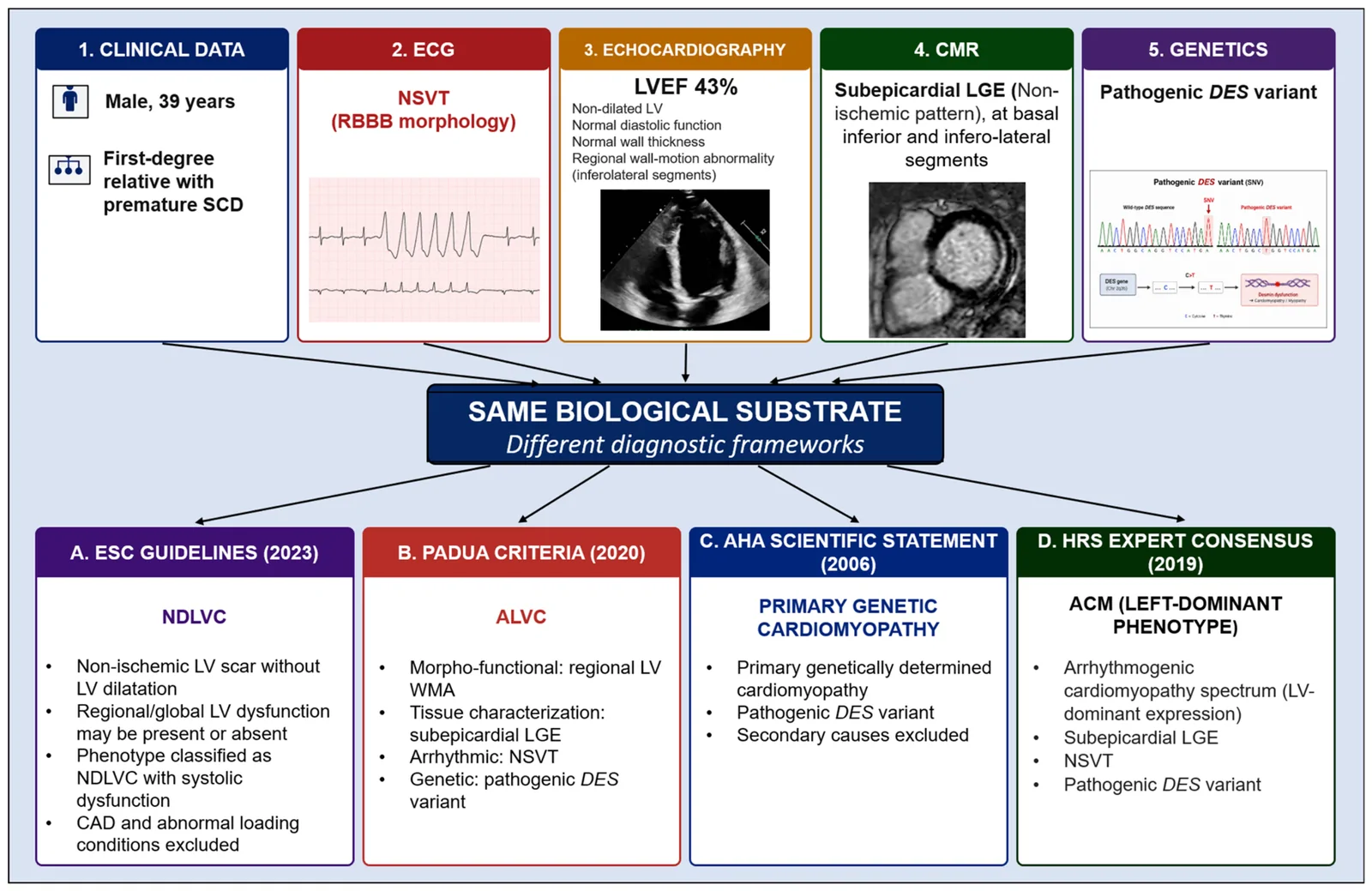
**Index case: taxonomic divergence in a single patient.** A 39-year-old man with a family history of premature sudden cardiac death, NSVT with right bundle branch block morphology, a non-dilated LV, mildly reduced LVEF (43%), regional inferolateral wall motion abnormalities, subepicardial basal inferior and inferolateral LGE, preserved RV structure and function, and a pathogenic *DES* variant is classified differently across four major frameworks. (A) ESC 2023 [1]: NDLVC with systolic dysfunction based on non-ischemic LV scarring in the absence of LV dilatation or an alternative loading or ischemic cause. (B) Padua Criteria [2,3]: Left-dominant arrhythmogenic left ventricular cardiomyopathy (ALVC), supported by LV morpho-functional, tissue characterization, arrhythmic, and genetic criteria. (C) AHA 2006 [5]: Primary cardiomyopathy of genetic etiology without a fitting phenotypic category for this non-dilated, non-hypertrophic presentation. (D) HRS 2019 [4]: Left-dominant arrhythmogenic cardiomyopathy within the broader ACM spectrum. Abbreviations: ACM, arrhythmogenic cardiomyopathy; AHA, American Heart Association; ALVC, arrhythmogenic left ventricular cardiomyopathy; CAD, coronary artery disease; CMR, cardiac magnetic resonance; *DES*, desmin; ECG, electrocardiogram; ESC, European Society of Cardiology; HRS, Heart Rhythm Society; LGE, late gadolinium enhancement; LV, left ventricle; LVEF, left ventricular ejection fraction; NDLVC, non-dilated left ventricular cardiomyopathy; NSVT, non-sustained ventricular tachycardia; RBBB, right bundle branch block; RV, right ventricle; SCD, sudden cardiac death; SNV, single-nucleotide variant; WMA, wall motion abnormality.

This prototypical patient illustrates the taxonomic challenge at the core of this review: the same clinical and biological substrate receives different diagnostic or classificatory labels across four contemporary frameworks (Figure 1).

The same substrate can therefore carry different diagnostic labels without any underlying biological contradiction, underscoring a fundamental unresolved issue in contemporary cardiomyopathy classification. In this context, this review examines whether NDLVC represents a distinct biological disease entity or rather a shared phenotypic expression of multiple underlying cardiomyopathies and discusses the implications of this distinction for risk stratification, genetic counseling, and therapeutic decision making.

## 2. Classification Frameworks over Time

**Figure 2:**
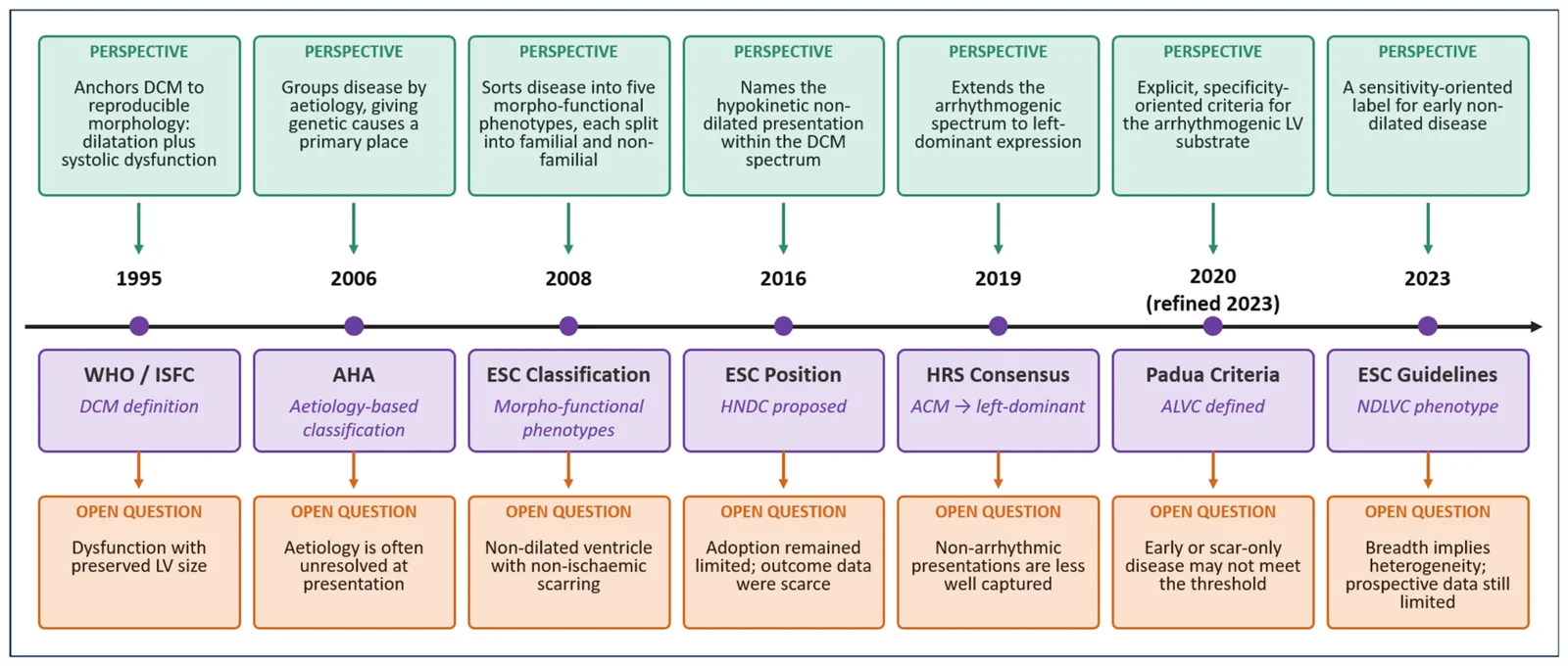
**Classification frameworks over time and their respective vantage points.** Seven frameworks are shown in chronological order: the 1995 WHO/ISFC definition, the 2006 AHA Scientific Statement, the 2008 ESC morpho-functional classification, the 2016 ESC Position Statement, the 2019 HRS consensus, the 2020 Padua Criteria with their 2023 European Task Force refinement, and the 2023 ESC Guidelines. For each framework, the upper card summarizes the perspective it contributes, whereas the lower card summarizes the question that remains open; these residual uncertainties reflect current gaps in evidence rather than contradictions between systems. Classification schemes specific to hypertrophic cardiomyopathy are deliberately not considered here, as they lie outside of the scope of this review, which concerns the non-dilated, non-hypertrophic left ventricular phenotype. Abbreviations: ACM, arrhythmogenic cardiomyopathy; AHA, American Heart Association; ALVC, arrhythmogenic left ventricular cardiomyopathy; DCM, dilated cardiomyopathy; ESC, European Society of Cardiology; HNDC, hypokinetic non-dilated cardiomyopathy; HRS, Heart Rhythm Society; LV, left ventricle; NDLVC, non-dilated left ventricular cardiomyopathy; WHO/ISFC, World Health Organization/International Society and Federation of Cardiology.

Over the last three decades, cardiomyopathy classifications have evolved in response to changing clinical needs and advances in disease understanding. Each framework reflects a distinct conceptual perspective prioritizing different diagnostic and clinical objectives rather than competing interpretations of the same condition. The World Health Organization/International Society and Federation of Cardiology (WHO/ISFC) classification anchored dilated cardiomyopathy (DCM) to morphological criteria, requiring left ventricular dilatation and systolic dysfunction [6], thereby excluding patients with systolic dysfunction despite preserved ventricular dimensions. The 2006 AHA Scientific Statement instead emphasized etiology, giving genetic disease a primary place [5], although etiology is often unresolved at presentation. The 2008 ESC classification reorganized cardiomyopathies by morpho-functional phenotype into hypertrophic, dilated, restrictive, arrhythmogenic right ventricular, and unclassified categories, each divided into familial and non-familial forms, while setting ion channel disorders aside [7]. As a taxonomy rather than a diagnostic framework, it offered no category for a non-dilated ventricle with non-ischemic scarring. The 2016 ESC Position Statement revised the definition of DCM, introducing hypokinetic non-dilated cardiomyopathy (HNDC) and formalizing intermediate and genotype-positive, phenotype-negative states for family screening, although HNDC saw limited subsequent adoption [8]. In parallel, the 2019 HRS consensus recognized left-dominant forms of arrhythmogenic cardiomyopathy [4], while the 2020 Padua Criteria provided explicit criteria for arrhythmogenic left ventricular involvement [2], later refined by the 2023 European Task Force report published in 2024 [3]. These frameworks prioritize arrhythmic substrate and risk and may therefore incompletely capture early or predominantly non-arrhythmic presentations. The 2023 ESC Guidelines subsequently adopted a phenotype-based classification and introduced NDLVC as one of five morpho-functional phenotypes [1]. This provides a diagnostic framework for early non-dilated disease but encompasses biologically heterogeneous presentations for which prospective data remain limited. The frameworks are therefore complementary rather than mutually exclusive, and their unresolved differences may reflect gaps in evidence more than genuine disagreement about biology. Their respective perspectives are summarized in Figure 2.

## 3. Rationale for the Introduction of NDLVC

**Figure 3:**
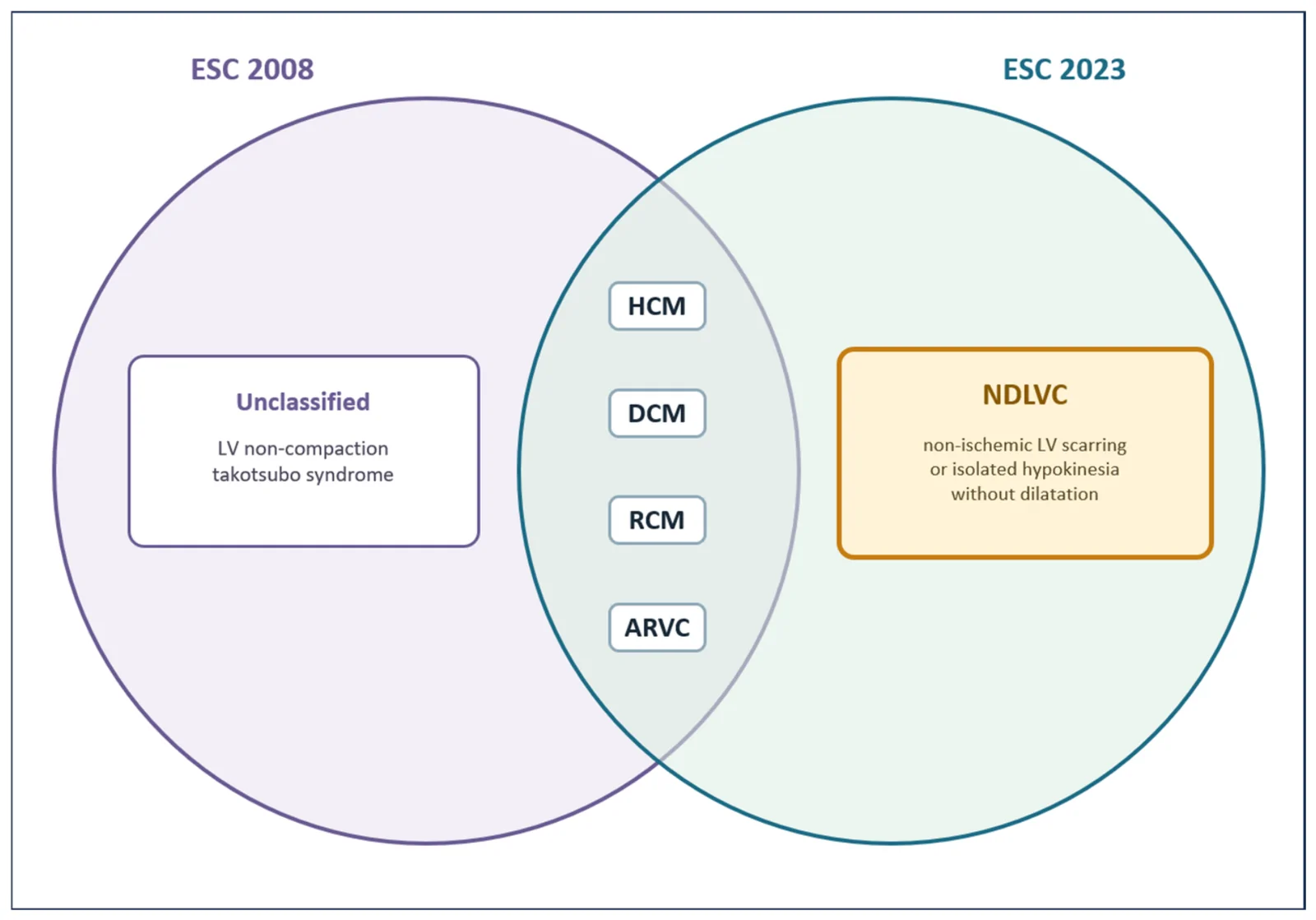
**Phenotypes of the 2008 and 2023 ESC classifications.** The two overlapping circles represent the phenotypes of each classification. Four phenotypes lie in the intersection and are shared by both: hypertrophic, dilated and restrictive cardiomyopathy, and arrhythmogenic right ventricular cardiomyopathy. The two classifications differ in one category each, shown in the non-overlapping regions. The 2008 classification included an unclassified group comprising left ventricular non-compaction and takotsubo syndrome, which the 2023 Guidelines no longer retain as a distinct phenotype, non-compaction being regarded as a morphological trait that may accompany other phenotypes rather than a phenotype in itself. Conversely, the non-dilated left ventricular phenotype introduced in 2023, highlighted in amber, had no counterpart in 2008: a patient with this presentation could only be assigned to the unclassified group or left outside of the classification altogether. Abbreviations: ARVC, arrhythmogenic right ventricular cardiomyopathy; DCM, dilated cardiomyopathy; ESC, European Society of Cardiology; HCM, hypertrophic cardiomyopathy; LV, left ventricular; NDLVC, non-dilated left ventricular cardiomyopathy; RCM, restrictive cardiomyopathy.

**Figure 4:**
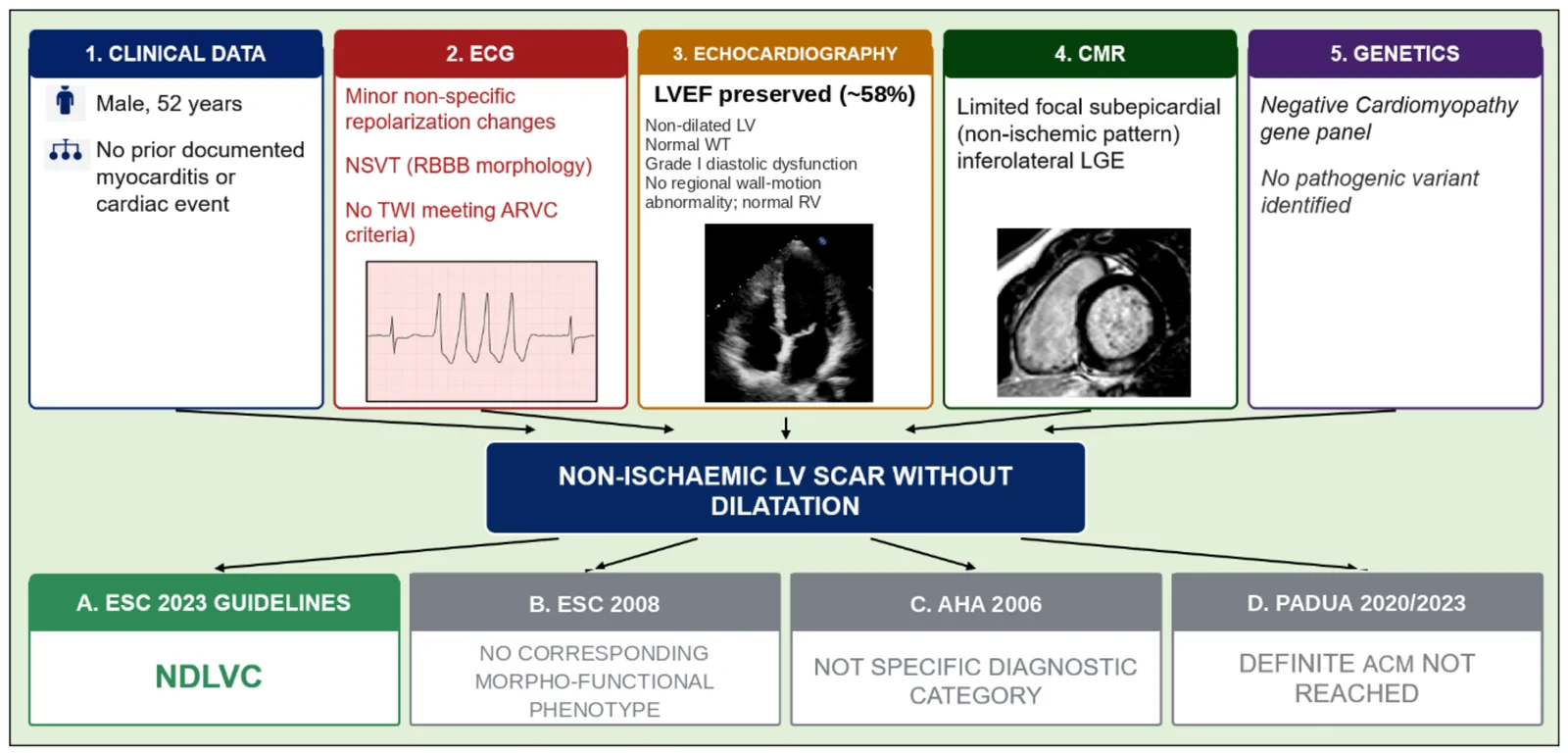
**Phenotypic classification of a patient with non-ischemic LV scarring without dilatation.** A 52-year-old man with preserved LVEF (~58%), a non-dilated LV of normal wall thickness, no regional wall motion abnormalities, normal RV, limited focal subepicardial inferolateral LGE, and NSVT with RBBB morphology, with negative genetic testing and no history of myocarditis. (A) 2023 ESC Guidelines [1]: NDLVC, based on non-ischemic LV scarring in the absence of LV dilatation. (B) 2008 ESC classification [7]: No corresponding phenotype. (C) 2006 AHA classification [5]: No specific phenotypic category for this presentation. (D) Padua Criteria and subsequent European Task Force revision [2,3]: Criteria insufficient for a definite diagnosis of ALVC/ACM; under the 2020 Padua Criteria, a pathogenic ACM gene variant is required in the absence of RV involvement, whereas the subsequent European Task Force revision no longer requires a pathogenic variant but the available structural and arrhythmic criteria remain insufficient for a definite diagnosis. This case illustrates how NDLVC may capture a myocardial phenotype that remains unclassified or incompletely classified by previous frameworks. Abbreviations: ACM, arrhythmogenic cardiomyopathy; AHA, American Heart Association; ALVC, arrhythmogenic left ventricular cardiomyopathy; ARVC, arrhythmogenic right ventricular cardiomyopathy; CMR, cardiac magnetic resonance; ECG, electrocardiogram; ESC, European Society of Cardiology; LGE, late gadolinium enhancement; LV, left ventricle; LVEF, left ventricular ejection fraction; NDLVC, non-dilated left ventricular cardiomyopathy; NSVT, non-sustained ventricular tachycardia; RBBB, right bundle branch block; RV, right ventricle; TWI, T-wave inversion; WT, wall thickness.

The 2008 ESC classification grouped cardiomyopathies by morpho-functional phenotype into hypertrophic, dilated, restrictive, and arrhythmogenic right ventricular cardiomyopathy, together with an unclassified category that included left ventricular non-compaction and takotsubo syndrome [7]. The 2023 ESC Guidelines retain those four phenotypes, no longer list an unclassified category, and introduce NDLVC [1]; the two schemes are compared in Figure 3. The introduction of NDLVC reflects an evolution toward phenotype-based classification and a simplification of previously overlapping terminology, addressing limitations of earlier morphological definitions [1]. CMR detects and characterizes myocardial abnormalities in patients without overt LV dilatation, while advanced echocardiography, including speckle tracking, may identify subclinical dysfunction [1,9]. Patients with mildly reduced LVEF, normal LV dimensions, and non-ischemic fibrosis, particularly subepicardial LGE, were previously difficult to classify despite clear myocardial disease. By providing a framework for these early phenotypes, NDLVC may capture patients who would not meet conventional DCM criteria, including asymptomatic variant carriers identified through family screening with only mild systolic dysfunction or subtle global longitudinal strain impairment [1,10]. Earlier identification may allow for timely arrhythmic risk stratification, longitudinal follow-up before overt remodeling, and cascade family screening [9,11,12,13,14,15]. Clinical studies suggest an intermediate prognostic profile, with adverse outcomes driven predominantly by heart failure events occurring more often than in comparator groups without cardiomyopathy but less often than in overt DCM, differences that may partly reflect disease stage and cohort heterogeneity [9,12,13,16]. Ventricular arrhythmias (VA) may nonetheless develop despite preserved LV size, and sustained ventricular tachycardia (VT) is a recognized presenting manifestation of left-dominant ALVC, sometimes preceding structural progression [9,12,14,15,17], which highlights the value of early identification when closer surveillance and, in selected cases, implantable cardioverter–defibrillator (ICD) therapy may be warranted [16,17]. As a practical illustration, consider a 52-year-old man (Figure 4) with preserved LVEF (~58%), a non-dilated left ventricle of normal wall thickness, grade I diastolic dysfunction, no regional wall motion abnormalities, a normal right ventricle, limited focal subepicardial inferolateral LGE, and non-sustained VT of RBBB morphology, with a negative gene panel and no prior myocarditis. Under conventional morphological definitions, these findings fit neither DCM, hypertrophic cardiomyopathy (HCM), nor arrhythmogenic right ventricular cardiomyopathy (ARVC). As Figure 4 shows, the frameworks do not converge: the 2020 Padua Criteria require a pathogenic ACM gene variant for ALVC in the absence of RV involvement; under the 2023 European Task Force revision, the two minor criteria present (limited LGE and NSVT of RBBB morphology) would likewise be insufficient; and the 2006 AHA scheme has no category for an isolated non-ischemic LV scar of undefined etiology. The 2023 ESC framework instead recognizes non-ischemic LV scarring without dilatation as NDLVC [1]. Once active or reversible causes are excluded, recognition of this phenotype may facilitate structured arrhythmic risk and family assessment.

## 4. Epidemiology

Precise prevalence estimates are limited by the phenotype’s definition only in 2023 and by heterogeneity among earlier cohorts; the ESC Guidelines define NDLVC but not its epidemiology. Current estimates, summarized in Table 1, are therefore derived from retrospective or single-center cohorts [9,10,13,15,18,19,20,21,22,23]. NDLVC is detected in approximately 2.5% of patients referred for stress perfusion or viability CMR, rising to 4.8% after exclusion of ischemic and specific etiologies, and appears more frequent in arrhythmia-predominant and tertiary care settings [9,10]. Pathogenic or likely pathogenic variants are identified in approximately 30–40% of patients, with substantial variation according to referral setting and testing strategy; *DSP*, *FLNC*, *LMNA*, and *PLN* predominate in specialized cohorts [10,18,22]. Non-ischemic LGE is present in 58.2% of patients in the largest consecutive CMR referral cohort reporting this measure, with higher proportions reported in arrhythmia referral populations [9,15]. VA are common but highly cohort-dependent [12,13], while atrial fibrillation appears more frequently than DCM among patients with reduced LVEF [21]. Progression to DCM reached 28.9% over a median follow-up of 77 months in one large series [23].

## 5. Genetics

The genetic architecture of NDLVC is heterogeneous and encompasses variants in genes encoding desmosomal proteins (e.g., *DSP*), cytoskeletal proteins (e.g., *FLNC*, *DES*), nuclear envelope proteins (e.g., *LMNA*), calcium handling proteins (e.g., *PLN*), as well as other disease-associated genes, including *TMEM43* and *RBM20* [1,24]. This architecture carries two opposing messages. On one hand, no gene is unique to NDLVC: the same variants underlie dilated and arrhythmogenic cardiomyopathy, and penetrance and variable expressivity determine which phenotype emerges, which argues for a shared phenotype [25,26,27,28]. On the other hand, NDLVC does not simply reproduce the genetic profile of DCM: pathogenic and likely pathogenic variants in pro-arrhythmogenic genes are considerably more frequent in NDLVC than in DCM, although no single genotype is specific to the phenotype [16,24]. A genetic profile that is shared in its components yet distinct in its distribution therefore does not adjudicate the question posed in this review; it sharpens it. Genotype–phenotype correlations and their implications for risk stratification are detailed in Table 2.

**Table 2:** **Genotype–phenotype correlations and risk stratification in NDLVC-associated genes**. Table constructed from published genotype–phenotype correlation studies. Risk levels are relative comparisons within NDLVC genetic etiology; genotype-specific late gadolinium enhancement patterns, with their main supporting cohort data, are detailed in Figure 5 and the accompanying text. Genes listed among high-risk genotypes in the 2023 ESC guidelines but not commonly reported with a non-dilated left ventricular presentation, such as *TMEM43*, are referred to in Section 8 but are not tabulated here. Dystrophin (*DMD*) is not tabulated as an NDLVC gene because the 2023 ESC guidelines assign it to the dilated and non-dilated phenotypes without a curated arrhythmogenic association, but it is retained in Figure 5 because extensive inferolateral subepicardial LGE is a recognized clue to dystrophinopathy. Abbreviations: ACM, arrhythmogenic cardiomyopathy; ALVC, arrhythmogenic left ventricular cardiomyopathy; CMR, cardiac magnetic resonance; DCM, dilated cardiomyopathy; *DES*, desmin; *DMD*, dystrophin; *DSP*, desmoplakin; ESC, European Society of Cardiology; *FLNC*, filamin C; LGE, late gadolinium enhancement; *LMNA*, lamin A/C; LVRR, left ventricular reverse remodeling; NDLVC, non-dilated left ventricular cardiomyopathy; *PLN*, phospholamban; *RBM20*, RNA-binding motif 20; RCM, restrictive cardiomyopathy; *TMEM43*, transmembrane protein 43; *TTN*, titin.

Gene	Typical Phenotype(s)	CMR Findings	Arrhythmic Risk, Prognosis, and Management
***DSP***	NDLVC, ALVC, left-dominant ACM	Subepicardial/ring-like inferolateral LGE	High arrhythmic risk; myocarditis-like injury episodes and adverse arrhythmic and heart failure outcomes [29,30]
***FLNC***	NDLVC, left-dominant ACM, DCM	Subepicardial/ring-like or mid-wall LGE	Very high arrhythmic risk with truncating variants; supports individualized risk assessment [1,31,32]
***LMNA***	DCM, NDLVC, conduction disease	Mid-wall septal LGE	Very high arrhythmic risk; early conduction disease; dedicated risk assessment [18,33]
***PLN***	NDLVC, DCM, biventricular ACM	Inferolateral to ring-like subepicardial LGE	High arrhythmic risk; p.Arg14del associated with an aggressive arrhythmic course [1,34,35]
***DES***	DCM, RCM, NDLVC, skeletal myopathy	Subepicardial/ring-like and/or mid-wall LGE	High arrhythmic risk; left-dominant or biventricular disease, conduction, and neuromuscular involvement [1,4,25,36]
***RBM20***	DCM, NDLVC	Often absent or limited LGE	High arrhythmic risk; rapid progression and electrical instability in selected patients [18,37,38]
***TTN***	DCM, NDLVC	Often absent or non-specific LGE	Arrhythmic risk less genotype-specific than *DSP*/*FLNC*/*LMNA*; truncating variants may increase ventricular arrhythmic risk; LVRR relatively frequent [1,18,37,39,40,41]

**Figure 5:**
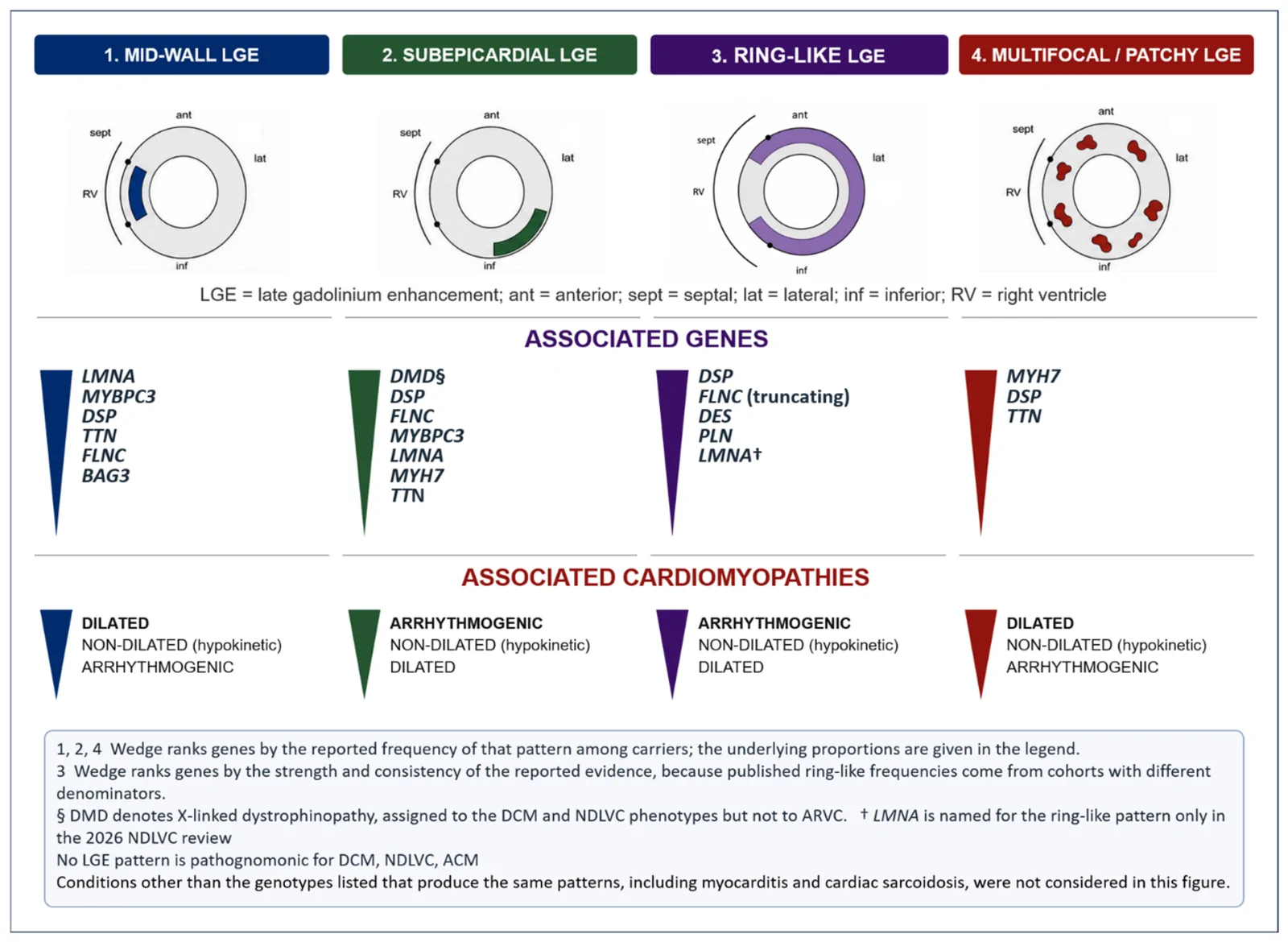
**CMR LGE patterns across the DCM–NDLVC–ACM spectrum.** Four representative non-ischemic late gadolinium enhancement (LGE) patterns are shown: (1) mid-wall septal linear LGE, (2) subepicardial LGE, (3) ring-like subepicardial LGE, and (4) multifocal patchy LGE. The anatomical distribution shown is representative rather than exclusive; mid-wall LGE is most commonly observed in the interventricular septum, whereas subepicardial LGE more frequently involves the inferolateral and lateral LV wall. The figure illustrates the substantial overlap of LGE patterns across dilated cardiomyopathy (DCM), non-dilated left ventricular cardiomyopathy (NDLVC), and arrhythmogenic cardiomyopathy (ACM). Genotype associations are presented as relative tendencies rather than disease-specific relationships; their position reflects reported associations or relative frequencies and does not imply diagnostic specificity. RV insertion point enhancement is shown separately because of its non-specific nature. Abbreviations: ACM, arrhythmogenic cardiomyopathy; ARVC, arrhythmogenic right ventricular cardiomyopathy; *BAG3*, BCL2-associated athanogene 3; CMR, cardiac magnetic resonance; DCM, dilated cardiomyopathy; *DES*, desmin; *DMD*, dystrophin; *DSP*, desmoplakin; *FLNC*, filamin C; LGE, late gadolinium enhancement; *LMNA*, lamin A/C; LV, left ventricle; *MYBPC3*, myosin binding protein C3; *MYH7*, myosin heavy chain 7; NDLVC, non-dilated left ventricular cardiomyopathy; *PLN*, phospholamban; RV, right ventricle; *TTN*, titin.

This shared but uneven genetic architecture has important implications for clinical risk stratification. First, the guideline-designated high-risk genotypes are *LMNA*, truncating *FLNC* and *TMEM43*, with reported annual sudden death rates of 5 to 10%, and *PLN*, *DSP,* and *RBM20*, with rates of 3 to 5%. *DES* does not appear in that table, but the guideline text on primary prevention of sudden death in NDLVC additionally names *DES* among the genotypes carrying a substantially higher rate of major arrhythmic events than other causes regardless of LVEF. These genotypes justify genotype-guided stratification, with variant-specific risk calculators documented in the guideline for *LMNA* and *PLN* p.Arg14del, while genotype-specific models for the other high-risk genes remain emerging [1,24]; their arrhythmic weight extends beyond LVEF and LGE burden [37,42]. Second, most patients remain genotype-negative. In them, no gene-specific tool applies, and guidelines default to DCM criteria for primary prevention ICD implantation [1,24], even though a negative genetic test has itself been associated with a lower risk of adverse remodeling [23,24]. Whether genotype-negative NDLVC is a distinct biological entity or reflects the current limits of sequencing, with additional heritability attributable to polygenic risk, non-coding variants, and epigenetic mechanisms, remains unresolved; it is precisely in this majority subgroup, where genetics is least informative, that CMR-based phenotyping and cascade family screening carry the greatest weight [1,24,37].

## 6. Multimodality Imaging

Multimodality imaging plays a central role in the phenotypic characterization and risk stratification of NDLVC [1,9,43,44]. Echocardiography is the first-line imaging modality, providing assessment of LVEF, LV dimensions, regional wall motion abnormalities, and global longitudinal strain (GLS) [1,8]. Speckle tracking strain may detect subtle myocardial dysfunction before a decline in LVEF becomes apparent. CMR complements echocardiography by providing more accurate and reproducible assessment of LV volumes, wall thickness, LV mass, and systolic function, which may reclassify patients across cardiomyopathy phenotypes while serving as the reference standard for myocardial tissue characterization, including the detection and spatial distribution of fibrosis and edema [1,9,43]. LGE identifies fibrosis, and its pattern and distribution provide important etiological clues [15,45,46]. Beyond diagnosis, LGE presence and distribution provide important prognostic information, with LGE associated with VA and mortality [15,34,47]. Parametric mapping further complements this evaluation: native T1 may detect diffuse interstitial abnormalities beyond focal LGE and has been associated with adverse outcomes in non-ischemic cardiomyopathy [43,48]. Fatty replacement, reported in 20 to 30% of carriers of pathogenic *DSP* or *FLNC* variants and best characterized with dedicated fat-sensitive CMR sequences, typically involves the inferolateral wall and has been associated with ventricular ectopy and sustained ventricular tachycardia [24]. T2 mapping may identify myocardial edema and may support the assessment of active myocardial inflammation, although findings are not disease-specific [43]. In non-ischemic cardiomyopathy cohorts, extracellular volume (ECV) ≥ 30% has shown incremental prognostic value for VA beyond LGE and LVEF [43,49]; in that same cohort, however, neither native T1 nor global longitudinal strain retained an independent association with arrhythmic events after adjustment for LVEF [49]. Feature tracking strain identifies subclinical dysfunction and retains independent prognostic value despite preserved LVEF [50]. CMR also plays a central role in differential diagnosis, as CMR tissue characterization may help identify features suggestive of active inflammation and characterize the distribution of myocardial fibrosis, thereby contributing to the differential diagnosis of genetic cardiomyopathy and inflammatory phenocopies, including myocarditis, sarcoidosis, and Chagas disease. These findings should not be interpreted as disease-specific in isolation [9,45]. For arrhythmic risk stratification, integrating LGE extent and localization (e.g., ring-like pattern), ECV, and genotype may provide incremental information beyond LVEF alone [15,31]. Electroanatomic mapping identifies the arrhythmogenic substrate in refractory VT, although its role in NDLVC remains largely confined to patients undergoing catheter ablation [12,51]. Imaging is therefore indispensable but, in isolation, cannot fully discriminate between disease entities; accurate diagnosis and risk stratification require integration with genetic testing and longitudinal follow-up [1,43,52]. Cardiac CT may provide complementary information in selected patients, particularly when fibro-fatty myocardial replacement is suspected in the context of arrhythmogenic cardiomyopathy, whereas CMR remains the preferred modality for comprehensive tissue characterization and assessment of myocardial fibrosis and inflammation [53]. Notably, although LGE patterns show characteristic associations, they are not disease-specific, and a degree of overlap has been described across the DCM–NDLVC–ACM spectrum, mirroring the shared genetic architecture of these cardiomyopathies [1,2,3]. Genotype–LGE associations have nevertheless been described, including extensive subepicardial or ring-like scarring in desmoplakin cardiomyopathy [54]. Conversely, some genotype–phenotype associations may be characterized by absent or non-specific LGE, as reported for *RBM20*- and *TTN*-associated cardiomyopathy, underscoring that the absence of a characteristic LGE pattern does not exclude a genetic substrate [38,40]. Ring-like left ventricular scar has also been associated with increased arrhythmic risk in selected cohorts, although its prognostic value is not genotype-specific [55]. Representative non-ischemic patterns are summarized in Figure 5.

## 7. Disease or Phenotype?

**Figure 6:**
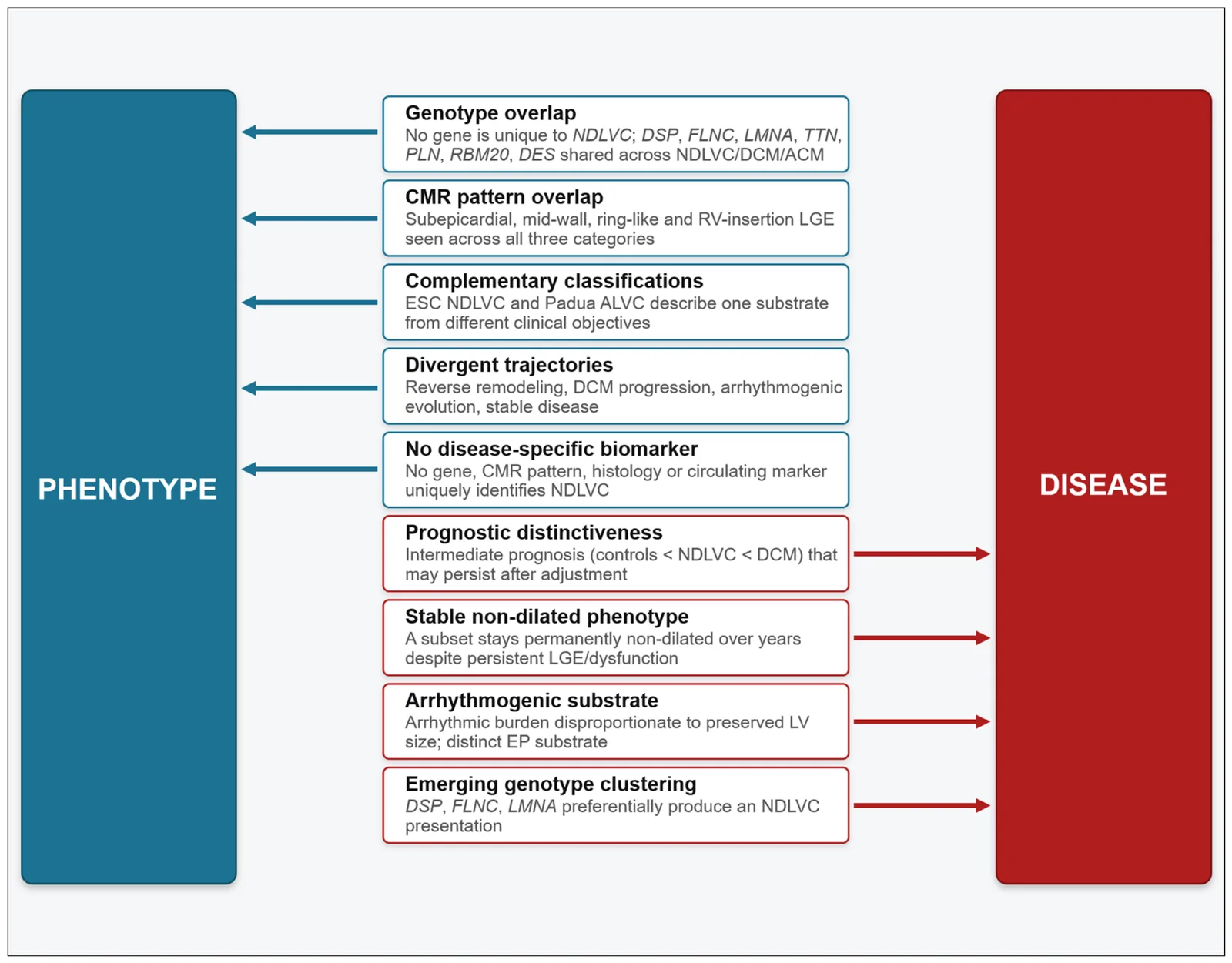
What weighs toward phenotype versus disease. Each central element is a line of evidence in the NDLVC debate; the arrow’s direction indicates whether that element weighs toward interpreting NDLVC as a shared morpho-functional phenotype (**left**) or as a distinct disease entity (**right**). The distinct genotypic setting element refers to the higher frequency of pathogenic variants in pro-arrhythmogenic genes reported in NDLVC than in DCM and to the frequency of guideline-designated high-risk genotypes among genotype-positive patients. In the prognostic element, the notation controls NDLVC; DCM denotes the reported ordering of adverse event rates, and RV insertion point LGE is abbreviated as RV insertion. The overlapping statements shown in the figure refer to reported occurrence and are deliberately summaries; as detailed in Table 3 and Figure 7, the strength of the association with ACM varies substantially between genes, and RV insertion point enhancement, although seen across these phenotypes, is excluded from the ACM diagnostic criteria as non-specific and accordingly placed outside of the ACM circle in Figure 7. See Section 7.1 and Section 7.2. Abbreviations: ACM, arrhythmogenic cardiomyopathy; ALVC, arrhythmogenic left ventricular cardiomyopathy; CMR, cardiac magnetic resonance; DCM, dilated cardiomyopathy; *DES*, desmin; *DSP*, desmoplakin; EP, electrophysiological; ESC, European Society of Cardiology; *FLNC*, filamin C; LGE, late gadolinium enhancement; *LMNA*, lamin A/C; LV, left ventricle; NDLVC, non-dilated left ventricular cardiomyopathy; *PLN*, phospholamban; *RBM20*, RNA-binding motif 20; RV, right ventricle; *TTN*, titin.

The question posed in the subheading extends beyond semantics, as its answer bears on risk stratification, genetic counseling, family screening, and management. The ESC 2023 classification provides a valuable clinical framework, but the biological significance of the phenotype remains incompletely defined, and current evidence is compatible with both interpretations without conclusively supporting either. The lines of evidence favoring each are discussed below and summarized in Figure 6.

**Figure 7:**
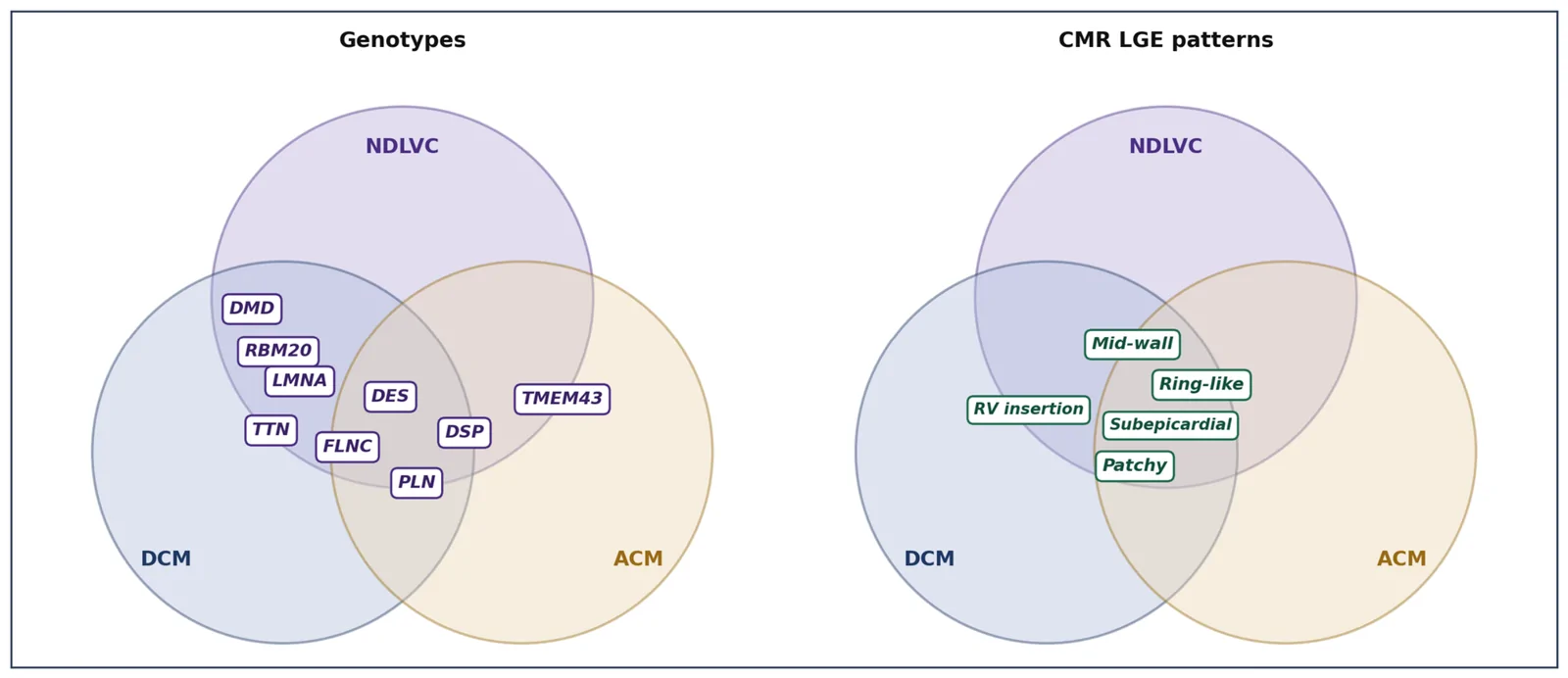
Neither genotype (**left panel**) nor CMR LGE pattern (**right panel**) is specific to NDLVC, DCM, or ACM. Genes and LGE patterns are positioned according to their reported occurrence across phenotypes; displacement within a circle indicates relative frequency and does not imply exclusivity or diagnostic specificity. Mid-wall, subepicardial, ring-like, and patchy LGE patterns overlap across the DCM–NDLVC–ACM spectrum, whereas RV insertion point enhancement is shown outside of the ACM circle because it is non-specific and excluded from ACM diagnostic criteria [2,3]. In the genotype panel, ACM circle membership reflects the strength of evidence for an arrhythmogenic cardiomyopathy phenotype rather than arrhythmic risk; therefore, guideline-designated high-risk genotypes such as *LMNA* and *RBM20* may lie outside of the ACM circle [1,24]. *TMEM43* is positioned at the margin of the NDLVC circle because it is not commonly reported with a non-dilated presentation [1]. Associations represent reported patterns rather than deterministic or diagnostic relationships. Abbreviations: ACM, arrhythmogenic cardiomyopathy; CMR, cardiac magnetic resonance; DCM, dilated cardiomyopathy; *DES*, desmin; *DMD*, dystrophin; *DSP*, desmoplakin; *FLNC*, filamin C; LGE, late gadolinium enhancement; *LMNA*, lamin A/C; NDLVC, non-dilated left ventricular cardiomyopathy; *PLN*, phospholamban; *RBM20*, RNA-binding motif 20; RV, right ventricular; *TMEM43*, transmembrane protein 43; *TTN*, titin.

**Table 3:** **Elements weighing toward NDLVC as a shared phenotype.** Abbreviations: ACM, arrhythmogenic cardiomyopathy; ALVC, arrhythmogenic left ventricular cardiomyopathy; CMR, cardiac magnetic resonance; DCM, dilated cardiomyopathy; *DES*, desmin; *DSP*, desmoplakin; ESC, European Society of Cardiology; *FLNC*, filamin C; LGE, late gadolinium enhancement; *LMNA*, lamin A/C; NDLVC, non-dilated left ventricular cardiomyopathy; *PLN*, phospholamban; *RBM20*, RNA-binding motif 20; RV, right ventricular; *TTN*, titin.

Element	Domain	What the Evidence Shows
Genotype overlap	Genetics	No gene is unique to NDLVC; *DSP*, *FLNC*, *LMNA*, *TTN*, *PLN*, *RBM20*, and *DES* overlap with DCM and with selected forms of ACM/ALVC, although the strength of curated evidence for the arrhythmogenic phenotype varies substantially between genes [10,18,19,32,33,34,35,38,39,53,55].
CMR/LGE phenotype overlap	Imaging	No LGE pattern is pathognomonic; subepicardial, mid-wall, ring-like, and RV insertion point patterns are seen across NDLVC, DCM, ACM, myocarditis, and sarcoidosis, although RV insertion point enhancement is non-specific and is not a diagnostic ACM pattern [15,43,56,57].
Complementary classifications	Taxonomy	ESC NDLVC and Padua ALVC may capture overlapping clinical substrates from different diagnostic perspectives; taxonomic disagreement does not necessarily imply biological disagreement [1,2,3,4].
Divergent trajectories	Natural history	NDLVC evolves along several paths (reverse remodeling, DCM progression, arrhythmogenic evolution, stable disease), consistent with a phenotypic umbrella [12,13,19,20,23,38].
No disease-specific biomarker	Biomarkers	No currently validated genetic, imaging, histological, or circulating biomarker uniquely identifies NDLVC [1,3,13,15,16,17,29,43,46,48,49,51].

### 7.1. The Case for “Phenotype”

Several converging observations support the interpretation of NDLVC as a shared morpho-functional phenotype rather than a distinct disease entity. No currently validated genetic variant, CMR pattern, histopathological finding, or circulating biomarker is unique to NDLVC, while substantial overlap exists with DCM and ACM, and longitudinal studies describe heterogeneous clinical trajectories [1,3,9,11,12,19,37]. These lines of evidence are summarized in Table 3.

#### 7.1.1. Genotype Overlap and CMR/LGE Phenotype Overlap

As discussed above, no gene is unique to NDLVC: pathogenic variants in *DSP*, *FLNC*, *LMNA*, *TTN*, *PLN*, *RBM20*, and *DES* overlap with DCM and with selected forms of ACM/ALVC, although the strength of the curated evidence for the arrhythmogenic phenotype varies substantially between genes, illustrating marked pleiotropy and variable expression [10,18,19,32,33,34,35,38,39,53,56,57]. The CMR phenotype is equally non-specific: subepicardial, mid-wall, and ring-like LGE occur across NDLVC, DCM, ACM, myocarditis, and sarcoidosis, whereas RV insertion point LGE, although frequently observed in these phenotypes, is explicitly excluded from the ACM diagnostic criteria as non-specific and is therefore not an ACM pattern [2,3], while T1, T2, and ECV mapping reflect tissue composition rather than disease-specific mechanisms [15,43]. Imaging findings must therefore be interpreted alongside clinical evaluation, genetic testing, and longitudinal follow-up. The genotype and LGE overlap is illustrated in Figure 7. Even the ring-like pattern, the most phenotype-suggestive of all, is not NDLVC-specific; NDLVC is only the second most prevalent phenotype among patients with a ring-like left ventricular scar, accounting for 37% of such cases [24].

#### 7.1.2. Classification Overlap

The discrepancy between the ESC NDLVC phenotype and the Padua definition of ALVC reflects different diagnostic objectives rather than different biology: the ESC classification emphasizes early phenotypic recognition and the Padua Criteria an arrhythmogenic substrate, so the same patient may satisfy one or both without biological contradiction [1,2,3,4].

#### 7.1.3. Disease Continuum

Longitudinal studies describe divergent trajectories, including reverse remodeling, progression toward DCM, persistent non-dilated disease, and predominantly arrhythmogenic evolution [12,13,19,20,23,38], a heterogeneity that favors interpretation of NDLVC as a phenotypic umbrella pending prospective confirmation.

#### 7.1.4. Absence of Disease-Specific Biomarkers

No currently validated biomarker across the genetic, imaging, histological, or circulating domains has been shown to uniquely identify NDLVC; all overlap extensively with DCM, ACM, and inflammatory cardiomyopathies [1,3,13,15,16,17,29,43,46,48,49,51]. Tissue characterization is moreover method-dependent rather than absolute: electroanatomic mapping may reveal substrate abnormalities that are not fully captured by LGE-CMR, particularly when fibrosis is intramural, subepicardial, or focal [12,24,51]. The technique-dependent nature of tissue characterization further limits its use as a disease-defining feature, and current evidence therefore favors interpretation as a shared morpho-functional phenotype.

### 7.2. The Case for “Disease”

The alternative view holds that NDLVC is a distinct entity, supported by four observations summarized in Table 4 [9,12,13,17,18,20,23,53]: an intermediate prognosis between populations without cardiomyopathy and overt DCM [9,12,13]; a subset remaining persistently non-dilated despite prolonged follow-up, with VA occurring despite preserved ventricular dimensions [9,13,17,20,23]; and preferential association of *DSP*, *FLNC*, and *LMNA* variants with a non-dilated presentation, suggesting disease stage specificity in selected genetic backgrounds [32,33,53].

Nevertheless, none of these findings is conclusive. Prognostic differences may reflect disease stage rather than distinct biology, stable phenotypes may represent incomplete penetrance, and genotype clustering may identify subsets within a broader cardiomyopathy spectrum. Consequently, current evidence remains insufficient to establish whether NDLVC should be regarded as a distinct disease or as a shared morpho-functional phenotype.

## 8. Clinical Implications

Regardless of how NDLVC is ultimately interpreted, patients require comprehensive evaluation and longitudinal follow-up [1,8]. As part of the initial evaluation, secondary and potentially reversible causes that may mimic an NDLVC phenotype should be systematically considered and excluded when clinically appropriate, including inflammatory/infectious, autoimmune/systemic, toxic, alcohol-related, and tachyarrhythmia-mediated causes. Importantly, alcohol exposure should be systematically assessed, including screening for unhealthy alcohol use, and complete abstinence should be recommended in patients with suspected alcohol-related myocardial disease or substantial/unhealthy alcohol consumption. Because current systems rely on different criteria, the same patient may be classified and risk-stratified differently, reflecting complementary objectives rather than biological contradiction; management should therefore be guided by disease trajectory rather than the diagnostic label. Recognizing NDLVC as a phenotype helps ensure that non-ischemic scars previously labeled post-myocarditis fibrosis or fibrosis of undetermined significance (Figure 4) are not overlooked. Management integrates multimodality imaging, genetic evaluation, family screening, arrhythmic risk stratification, and guideline-directed heart failure therapy. Echocardiography and CMR are central to diagnosis [1,43], CMR providing tissue characterization, LGE quantification, and assessment of RV involvement; repeat imaging should be individualized according to phenotype, genotype, severity, and evidence of progression or reverse remodeling [1,38]. Genetic testing with counseling is recommended in all patients because identifying a pathogenic variant informs prognosis, family screening, and arrhythmic risk stratification [1,10,37], and arrhythmic risk assessment should include ambulatory ECG monitoring, with exercise testing according to context [1,37]. Features that may support ICD consideration include pathogenic variants in the guideline-designated high-risk genes (*LMNA*, truncating *FLNC*, *TMEM43*, *PLN*, *DSP*, *RBM20*), while *DES*-associated disease warrants individualized consideration on the basis of its recognized arrhythmic risk, together with LVEF ≤ 35%, extensive LGE, non-sustained ventricular tachycardia, and syncope [1,12,15,37]. In carriers of a pathogenic or likely pathogenic variant in one of these genes with LVEF above 35%, an ICD should be considered when additional risk factors, defined as syncope or LGE on CMR, are present and may be considered in their absence; an ICD may likewise be considered without a high-risk genotype when those additional risk factors are present [1]. Ring-like LGE has been associated with increased arrhythmic risk in selected cohorts [55], and a 5-year NDLVC-specific risk score for major arrhythmic events has recently been derived in a two-center cohort and externally validated in an independent multicenter European cohort [58]. Heart failure should be managed with guideline-directed therapy and cardiac resynchronization considered in eligible symptomatic patients in sinus rhythm with LVEF ≤ 35%, left bundle branch block, and QRS complex duration ≥ 130 ms, in accordance with the heart failure and pacing guidelines adopted by reference in the 2023 ESC cardiomyopathy document [1]. Decisions should integrate phenotype, genotype, and overall clinical context rather than any single marker in isolation. Recognition of phenocopies is integral to this process, as acute myocarditis, cardiac sarcoidosis, tachycardia-induced, toxic, and peripartum cardiomyopathy may all present with a non-dilated phenotype and pathogenic DSP variants produce myocarditis-like hot-phase episodes that closely mimic acute myocarditis; myocardial inflammation documented through CMR and/or endomyocardial biopsy has been associated with increased major arrhythmic risk in selected NDLVC cohorts [58].

## 9. Biological Trajectories

**Figure 8:**
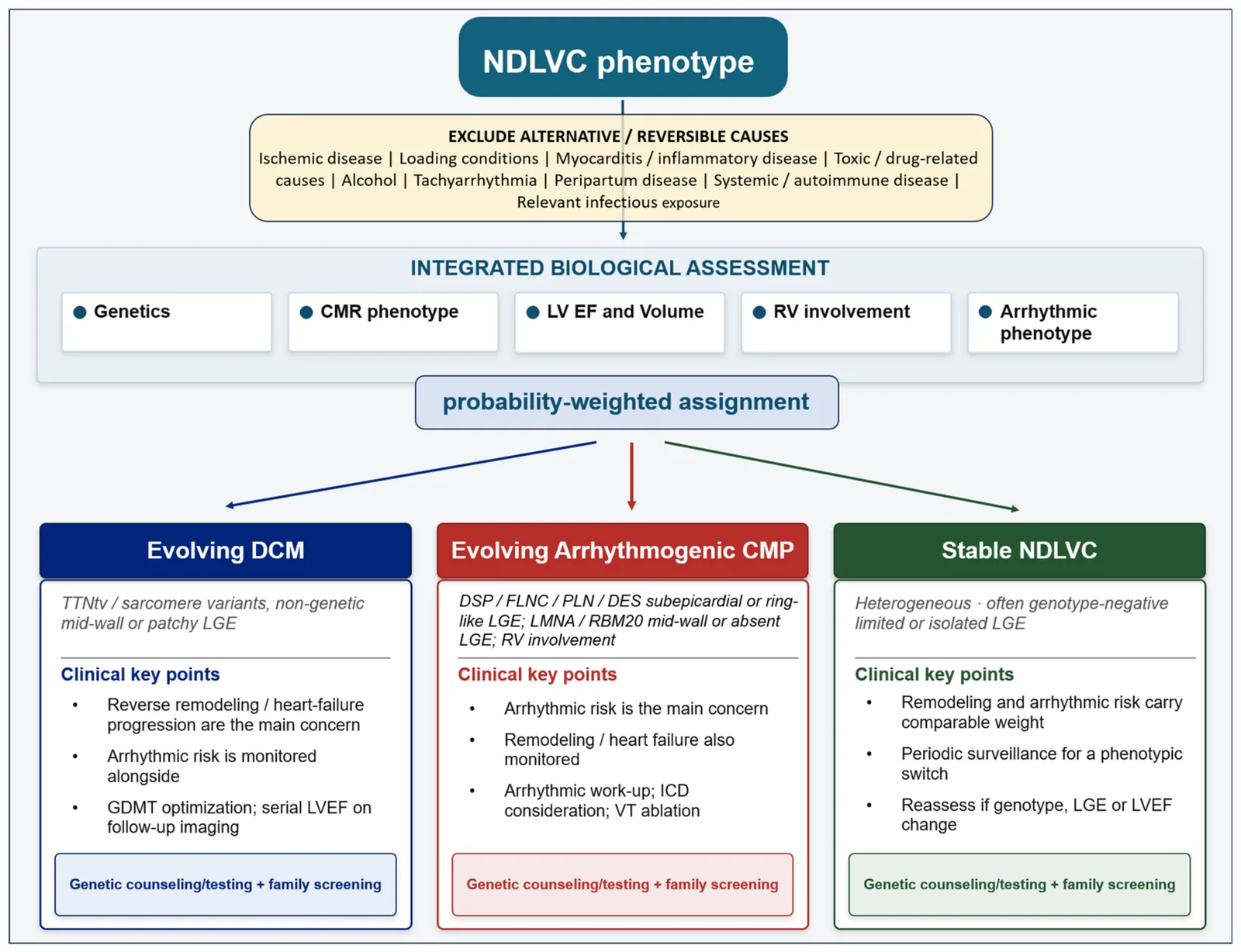
From the NDLVC phenotype to a probable biological trajectory. Proposed conceptual framework illustrating how integration of genotype, multiparametric CMR tissue characterization, including LGE pattern and burden, native T1, T2, and ECV mapping, longitudinal left ventricular remodeling, right ventricular involvement, and arrhythmic phenotype may estimate the most probable biological trajectory of patients presenting with NDLVC. Alternative and potentially reversible secondary causes should be considered and excluded when clinically appropriate before attributing the phenotype to a primary cardiomyopathy. The integrated assessment yields a probability-weighted assignment, that is, a qualitative judgement of which trajectory a given patient is most likely to be following, which serves as a first approximation of the conceptual trajectory rather than a calculated probability. Three conceptual trajectories are proposed, evolving DCM, evolving arrhythmogenic cardiomyopathy, and stable NDLVC, each associated with different follow-up priorities. Within the arrhythmogenic trajectory, the associated late gadolinium enhancement pattern differs by genotype, being subepicardial or ring-like for *DSP*, *FLNC*, *PLN*, and *DES* and mid-wall or absent for *LMNA* and *RBM20*. Genetic testing and family screening apply across all trajectories. This framework is hypothesis-generating and requires prospective validation. Abbreviations: CMP, cardiomyopathy; CMR, cardiac magnetic resonance; DCM, dilated cardiomyopathy; *DES*, desmin; *DSP*, desmoplakin; ECV, extracellular volume; EF, ejection fraction; *FLNC*, filamin C; GDMT, guideline-directed medical therapy; ICD, implantable cardioverter-defibrillator; LGE, late gadolinium enhancement; *LMNA*, lamin A/C; LV, left ventricle; LVEF, left ventricular ejection fraction; NDLVC, non-dilated left ventricular cardiomyopathy; *PLN*, phospholamban; *RBM20*, RNA-binding motif 20; RV, right ventricle; *TTNtv*, titin truncating variant; VT, ventricular tachycardia.

We propose a conceptual framework to explain the biological heterogeneity of NDLVC. Rather than representing a validated classification, this model is intended as a hypothesis-generating framework that integrates genotype, multiparametric CMR tissue characterization, including LGE distribution and burden, native T1, T2, and ECV mapping, longitudinal left ventricular remodeling, right ventricular involvement, and arrhythmic phenotype to estimate the most probable biological trajectory of an individual patient. The assignment is probability-weighted in a qualitative sense, serving as a first approximation of the conceptual trajectory rather than a calculated probability. Three conceptual trajectories are proposed: evolving DCM, characterized predominantly by progressive ventricular remodeling and heart failure progression; evolving arrhythmogenic cardiomyopathy, characterized by increasing arrhythmic substrate and risk; and stable NDLVC, in which the non-dilated phenotype persists over time with limited structural progression. These trajectories should be regarded as conceptual biological constructs rather than fixed categories, with genetic testing and cascade family screening considered in all patients [1] (Figure 8).

The framework also reframes the apparent discrepancy between different classification systems; rather than representing competing classifications, these systems may describe different segments of the same biological continuum. The remaining challenge is therefore biological rather than taxonomic, and future classifications will likely move beyond morphology by integrating genotype, multiparametric CMR, longitudinal trajectory, molecular biomarkers, and multiomics approaches.

## 10. Limitations and Gaps in Evidence

Several limitations temper the conclusions of this review and define the current gaps in evidence.

The evidence base is heterogeneous: most data are derived from retrospective, single-center, or specialist referral cohorts with variable inclusion criteria, so reported prevalence, genotype yield, and outcome estimates vary widely and are prone to referral and ascertainment bias [10,20,21]. This heterogeneity is compounded by the recency of the phenotype, which was defined only in 2023, so that its epidemiology and long-term outcomes remain incompletely characterized and much of the supporting literature predates the definition and was reinterpreted retrospectively [1,21]. A more fundamental constraint is that no genetic, imaging, histological, or circulating marker currently distinguishes NDLVC from dilated or arrhythmogenic cardiomyopathy at diagnosis; the overlap that is central to this review is also its principal evidentiary limitation [12,15]. The three-trajectory framework proposed here is correspondingly hypothesis-generating, as the proportion of patients in each trajectory, the baseline predictors of membership, and the prognostic consequences are unknown and untested prospectively [13,19,20]. Genotype adds less than might be expected, most patients remaining genotype-negative, so that current genomic tools capture only part of the heritable substrate [10,18]. Risk stratification, in turn, still relies largely on LVEF and LGE, yet a substantial share of arrhythmic events occurs outside of conventional high-risk categories, and emerging markers require prospective validation [15,58]. Addressing these gaps will require large, prospectively enrolled, deeply phenotyped cohorts with standardized imaging, comprehensive genetics, and long-term follow-up.

## 11. Future Perspectives

Several central questions remain to be addressed. An important priority is to determine whether biological trajectory can be predicted at diagnosis, with prospective studies integrating genotype, CMR phenotype, biomarkers, and longitudinal outcomes needed to better distinguish left ventricular reverse remodeling, DCM progression, and stable NDLVC [13,38,53]. Another key area is whether arrhythmic risk stratification can be refined beyond LVEF and LGE. An NDLVC-specific risk score has recently been derived and externally validated [58], while genotype-based approaches are also emerging. However, major arrhythmic events may still occur in patients who do not meet conventional high-risk criteria [58], highlighting the potential value of complementary markers such as mechanical dispersion, artificial intelligence ECG analysis, and radiomics, which remain investigational. Finally, the development of targeted therapies for selected subgroups, including anti-fibrotic, anti-arrhythmic, and gene- or RNA-based approaches, represents an area of growing interest. International registries with standardized phenotyping and data sharing will be important to further validate risk models and evaluate emerging therapeutic strategies.

## 12. Conclusions

NDLVC represents a clinically useful and conceptually complex phenotype in contemporary cardiology encompassing a broad spectrum of clinical and biological features. The evidence reviewed here suggests that its underlying substrates may be heterogeneous and that NDLVC may not necessarily correspond to a single genetic, CMR-defined, or prognostic entity. Rather, it may be viewed as a clinically useful morpho-functional framework that captures different biological substrates and stages of disease expression. Differences between the 2023 ESC Guidelines and other classification systems need not imply biological contradiction, as these systems were often developed for different diagnostic objectives. Three provisional trajectories (evolving DCM, evolving arrhythmogenic cardiomyopathy, and stable NDLVC) provide a working conceptual framework, but prospective validation in large, deeply phenotyped cohorts is indispensable before this framework can guide clinical decisions. The critical unresolved question is not “disease or phenotype?” but “which biological trajectory will each individual patient follow?” Answering this will require integration of genotype, multiparametric CMR, longitudinal biomarkers, and multi-omics data at the time of initial diagnosis.

## Figures and Tables

**Table 1:** **Estimated epidemiological parameters in NDLVC.** All estimates are derived from retrospective or single-center cohorts with heterogeneous inclusion criteria; prospective population-based data are lacking. Abbreviations: CMR, cardiac magnetic resonance; DCM, dilated cardiomyopathy; LGE, late gadolinium enhancement; LV, left ventricle; NDLVC, non-dilated left ventricular cardiomyopathy; RV, right ventricle; VA, ventricular arrhythmias.

Parameter	Estimated Value
**Prevalence in CMR-referred populations**	2.5% of patients referred for stress perfusion or viability CMR; 4.8% of the cohort analyzed after predefined exclusions [9]
**Prevalence in other clinical settings**	Higher in family screening and arrhythmia referral cohorts than in unselected populations [10,24]
**Pathogenic variant yield (comprehensive panel)**	~30–40% [10,18,22]
**LGE prevalence**	58.2% (64/110) in a consecutive CMR referral cohort; among LGE-positive patients, RV insertion point enhancement was most frequent (60.9%), followed by mid-wall (18.8%), subepicardial (17.2%), and patchy (9.4%) patterns [9]. Higher LGE prevalence has been reported in arrhythmia referral cohorts [9,15]
**VA prevalence at diagnosis**	Common but highly cohort-dependent; no reliable single prevalence range [12,13]
**Atrial fibrillation prevalence**	More frequent than in DCM among patients with reduced ejection fraction [21]
**LV dilatation/DCM progression rate**	28.9% over a median 77-month follow-up [23]

**Table 4:** **Elements weighing toward NDLVC as a distinct disease**. Abbreviations: DCM, dilated cardiomyopathy; *DSP*, desmoplakin; *FLNC*, filamin C; *LMNA*, lamin A/C; LV, left ventricular; NDLVC, non-dilated left ventricular cardiomyopathy; VA, ventricular arrhythmias.

Element	Domain	What the Evidence Shows
Prognostic distinctiveness	Prognosis	NDLVC carries an intermediate prognosis between controls and DCM that may persist after risk factor adjustment, arguing against it being merely early DCM [9,12,13].
Stable non-dilated phenotype	Natural history	A subset remains non-dilated during prolonged follow-up despite persistent myocardial abnormalities, suggesting the possibility of a biologically stable phenotype [9,13,20,23].
Arrhythmogenic substrate	Electrophysiology	VA may occur despite preserved LV dimensions, supporting the possibility of a distinct electrophysiological substrate not fully explained by LV size [9,13,17,20].
Emerging genotype clustering	Genetics	Selected genotypes, including *DSP*, *FLNC*, and *LMNA*, have been associated with non-dilated or left-dominant presentations in selected cohorts [32,33,53].

## Data Availability

The clinical images presented in this review are not publicly available because they are subject to institutional and privacy restrictions. They may be made available by the corresponding author upon reasonable request and subject to institutional authorization.

## Abbreviations

The following abbreviations are used in this manuscript:

**ACM**	Arrhythmogenic cardiomyopathy
**AHA**	American Heart Association
**ALVC**	Arrhythmogenic left ventricular cardiomyopathy
**ARVC**	Arrhythmogenic right ventricular cardiomyopathy
**BAG3**	BCL2-associated athanogene 3 gene
**CAD**	Coronary artery disease
**CMP**	Cardiomyopathy
**CMR**	Cardiac magnetic resonance
**DCM**	Dilated cardiomyopathy
**DES**	Desmin gene
**DMD**	Dystrophin gene
**DSP**	Desmoplakin gene
**ECG**	Electrocardiogram
**ECV**	Extracellular volume
**EF**	Ejection fraction
**EP**	Electrophysiological
**ESC**	European Society of Cardiology
**FLNC**	Filamin C gene
**GDMT**	Guideline-directed medical therapy
**GLS**	Global longitudinal strain
**HCM**	Hypertrophic cardiomyopathy
**HNDC**	Hypokinetic non-dilated cardiomyopathy
**HRS**	Heart Rhythm Society
**ICD**	Implantable cardioverter-defibrillator
**ISFC**	International Society and Federation of Cardiology
**LGE**	Late gadolinium enhancement
**LMNA**	Lamin A/C gene
**LV**	Left ventricular/left ventricle
**LVEF**	Left ventricular ejection fraction
**LVRR**	Left ventricular reverse remodeling
**MYBPC3**	Myosin binding protein C3 gene
**MYH7**	Myosin heavy chain 7 gene
**NDLVC**	Non-dilated left ventricular cardiomyopathy
**NSVT**	Non-sustained ventricular tachycardia
**PLN**	Phospholamban gene
**QRS**	QRS complex duration on the electrocardiogram
**RBBB**	Right bundle branch block
**RBM20**	RNA-binding motif protein 20 gene
**RCM**	Restrictive cardiomyopathy
**RV**	Right ventricular/right ventricle
**SCD**	Sudden cardiac death
**SNV**	Single-nucleotide variant
**TMEM43**	Transmembrane protein 43 gene
**TTN**	Titin gene
**TTNtv**	Titin truncating variant
**TWI**	T-wave inversion
**VA**	Ventricular arrhythmias
**VT**	Ventricular tachycardia
**WHO**	World Health Organization
**WMA**	Wall-motion abnormality
**WT**	Wall thickness
